# Alcohol and the Cardiovascular System: Molecular Mechanisms for Beneficial and Harmful Action

**Published:** 1997

**Authors:** Sam Zakhari

**Affiliations:** Sam Zakhari, Ph.D., is chief of the Biomedical Research Branch at the National Institute on Alcohol Abuse and Alcoholism, Bethesda, Maryland

**Keywords:** chronic AODE (alcohol and other drug effects), molecular interaction, biochemical mechanism, lipoproteins, platelets, blood coagulation, moderate AOD use, heavy AOD use, therapeutic drug effect, AODR (alcohol and other drug related) disorder, alcoholic cardiomyopathy, cardiac arrhythmia, hypertensive disorder, stroke, literature review, cell signaling

## Abstract

Alcohol can be beneficial or harmful to the cardiovascular system, depending on the amount consumed and the characteristics of the consumer. Of the numerous cellular and molecular mechanisms that are thought to explain the beneficial effects of moderate drinking, this article discusses four, involving (1) high density lipoproteins, (2) cellular signaling, (3) platelet function in blood clot formation, and (4) stimulation of blood clot dissolution. Although light-to-moderate drinking can protect against coronary artery disease, heavy alcohol consumption can damage the cardiovascular system, resulting in maladies such as heart muscle disorders, irregular heart rhythms, high blood pressure, and strokes. This article summarizes representative epidemiological and animal studies on these cardiovascular consequences of chronic heavy alcohol consumption and reviews mechanisms that have been suggested to explain alcohol’s effects.

Alcoholic beverages have been used—and abused—since the dawn of history. Although most people who choose to drink can limit their intake to a level that produces no harm to their health or to society, about 34 percent of the population drinks approximately 62 percent of all alcoholic beverages consumed. This chronic heavy drinking^1^ is a significant factor in the development of alcohol dependence, or alcoholism,^2^ and is associated with serious adverse health consequences, including negative effects on the cardiovascular system, such as heart muscle disorders (i.e., cardiomyopathy), heartbeat rhythm irregularities (i.e., arrhythmias), high blood pressure (i.e., hypertension), and strokes.

In contrast, moderate drinking^3^ has been known since ancient times to promote conviviality and decrease tension, anxiety, and self-consciousness; the possible physical health benefits of moderate drinking were noted long ago as well.^4^ In modern times, numerous epidemiological studies associate moderate alcohol consumption with protective health benefits (see table), including a lower risk of coronary artery disease (CAD) (see, for example, Boffetta and Garfinkel 1990). In the United States, where CAD accounts for about 450,000 deaths every year, some people advocate moderate drinking to reduce the risk of death from CAD. Moderate drinking is not risk free, however (see, for example, Criqui 1996), and careful analysis of the tradeoffs between its benefits and risks should be considered on an individual basis and across the life span (Dufour 1996; Zakhari and Wassef 1996).

This article first focuses on advances from biochemical research that have improved our understanding of alcohol’s beneficial effects on the cardiovascular system. Building on this foundation, the article next examines several specific consequences of long-term heavy alcohol consumption on the cardiovascular system.

## Biochemical Studies

Biochemical studies designed to address the mechanisms of alcohol’s effects on CAD risk have focused on factors that promote or protect against the abnormal narrowing of coronary arteries and the formation of fatty plaque deposits (i.e., atherogenesis),^5^ which leads to degenerative changes in the arterial walls and eventually to “hardening of the arteries” (i.e., atherosclerosis). Research using a genetically defined inbred strain of mice confirmed that alcohol can reduce arterial plaque formation, but the underlying mechanisms by which alcohol influences the several interdependent events leading to atherosclerosis (see, for example, Foegh and Virmani 1993) are not totally clear. In addition, it should be noted that some of these mechanisms work after repeated or chronic alcohol consumption (such as the effect on lipoproteins), but some start to act after a single drink (such as the effect on blood platelets) as described below. This article reviews findings on the following factors that have received research attention:

- Blood concentrations of high density and low density lipoproteins
- Cellular signaling
- Blood clot formation by cells known as platelets
- Blood clot dissolution through enzyme action.

### Alcohol’s Effects on High Density and Low Density Lipoprotein Levels

Major plasma lipids (i.e., the fatlike substances cholesterol and triglycerides) circulate as macromolecular complexes of fat molecules and proteins (i.e., lipoproteins). Of the several different types of lipoproteins present in the body, two are known to have particular relevance to the formation of arterial plaque deposits: high density and low density lipoproteins. In the simplest terms, high levels of high density lipoproteins (HDL) and low levels of low density lipoproteins (LDL) are desirable. Current recommendations advocate plasma HDL levels of at least 35 milligrams per deciliter (mg/dL) and LDL levels of less than 160 mg/dL in adults. Ideally, the LDL:HDL ratio should be between 2.5:1 and 4.5:1.

Chylomicrons and very low density lipoproteins (VLDL) are two other major classes of lipoproteins in the body. Chylomicrons, the largest lipoprotein particles, carry triglycerides consumed in food from the intestine to the blood, whereas the smaller VLDL particles are formed mainly in the liver and enter the blood circulation to transport cholesterol and triglycerides to peripheral tissues throughout the body. In the capillaries within fatty tissues and muscles, an enzyme known as lipoprotein lipase (LPL) breaks down triglycerides in both chylomicrons and VLDL to substances used in metabolism and energy storage (i.e., fatty acids and glycerol).

As LPL breaks down VLDL molecules, LDL particles are formed. LDL particles contain large concentrations of cholesterol and phospholipids. Some cholesterol is released to body tissues, where it plays an important role in maintaining cell membranes and synthesizing steroids such as sex hormones, corticosteroids, bile acids, and vitamin D. LDL cholesterol not delivered to tissues (about 60 percent) may be recaptured by special LDL receptors in the liver. If LDL molecules are not recaptured by the liver (e.g., because of a hereditary deficiency of LDL receptors), blood levels of cholesterol rise. Because accumulated cholesterol forms a major component of the characteristic fatty plaques that build up along artery walls in atherogenesis, high cholesterol levels often are associated with CAD.

Clinical studies have shown, however, that every 1-percent reduction in plasma cholesterol levels decreases the risk for CAD by 2 percent. HDL plays a key role in reducing cholesterol levels. Free cholesterol released from cells initially is incorporated into HDL by an enzyme called lecithin-cholesterol acyl transferase (LCAT), which changes the cholesterol to cholesteryl esters. To remove cholesterol from the circulation, the cholesteryl esters then are transported to LDL by cholesteryl ester transfer protein (CETP) for recapture by the LDL receptors in the liver. In addition, a newly discovered species of HDL called pre-high density lipoprotein binds with free cholesterol in a process known as reverse cholesterol transport, in which excess cholesterol is removed from body tissues, transported to the liver, and excreted in bile.

Biochemical studies examining alcohol’s effects on HDL are rooted in epidemiological studies that show an inverse relationship between plasma HDL cholesterol levels and CAD. Further epidemiological studies show an association between alcohol consumption and increased plasma HDL levels.^6^ A study by Linn and colleagues (1993) reported an increase of about 5 mg/dL in plasma HDL cholesterol levels after daily consumption of moderate amounts of alcohol.

The biochemical mechanisms for alcohol-induced increases in HDL levels are largely unknown, however. Several plausible mechanisms have been set forth, including stimulated production of two principal protein constituents of HDL (i.e., apolipoproteins A-I and A-II) in the liver (Välimäki et al. 1993). HDL levels also may be raised indirectly through increased activity of the enzyme LPL, which enhances the transfer of lipids and apolipoproteins from VLDL and chylomicrons. Nishiwaki and colleagues (1994) showed that LPL activity increased after 4 weeks of moderate alcohol consumption, and research by Välimäki and colleagues (1993) confirmed that LPL activity was linked to the consumption of alcohol by finding a significant decrease in LPL activity after alcoholic women abstained for 8 days.

In heavy drinkers, researchers have observed reduced activity of CETP, which also plays a role in regulating HDL cholesterol levels. Hannuksela and colleagues (1996) attributed this reduction to increased clearance of CETP from the blood rather than to a decrease in its cellular secretion. Such diminished CETP activity may maintain HDL levels by limiting the transfer of cholesteryl ester from HDL to LDL (Dreon and Krauss 1996). However, the role of CETP in increasing HDL is questionable, since this effect is inconsistent at low or moderate levels of alcohol consumption (e.g., Nishiwaki et al. 1994). Similarly, the observed increase in the activity of the enzyme LCAT may not play an important role in the alcohol-induced HDL increase (Nishiwaki et al. 1994).

**Table:** Effects of Moderate and Heavy Drinking

Beneficial Effects of Moderate Drinking	Harmful Effects of Heavy Drinking
Reduction of plaque deposits in arteries (i.e., atherosclerosis)	Increased risk for heart muscle disease (i.e., alcoholic cardiomyopathy)
Protection against blood clot formation, which protects against heart attack and atherosclerotic ischemic stroke	Increased risk for disturbed heart rhythm (i.e., arrhythmia)
Promotion of blood clot dissolution, which protects against heart attack and atherosclerotic ischemic stroke	Increased risk for high blood pressure Increased risk for hemorrhagic stroke

### Alcohol’s Effects on Cellular Signaling

Alcohol’s effects on plasma HDL and LDL levels apparently do not fully account for the reduction in CAD risk. In fact, Langer and colleagues (1992) concluded that no more than one-half of CAD risk reduction is associated with changes in HDL and LDL levels. The successful use of aspirin to decrease mortality from CAD points to the importance of factors other than cholesterol, because aspirin does not directly influence cholesterol metabolism. Such factors could include the cellular signaling that takes place in the inner lining (i.e., endothelium) of blood vessels. Indeed, investigation of endothelial cellular signaling is one of the most promising areas of research in alcohol-induced effects on the cardiovascular system.

Cells in the endothelium send out chemical signals that trigger an inflammatory process in response to the formation of fatty streaks in the arteries. This inflammatory process begins when LDL and immune cells (i.e., phagocytes) are trapped in the space beneath the interior lining of the artery walls (i.e., the subendothelial space). Factors released by phagocytes subsequently oxidize the LDL particles, leading to the activation of transcription factors such as NF-κB in endothelial cells. NF-κB is important in the development of atherosclerosis, because it regulates, at least in part, the copying of genetic information (i.e., transcription) of three cellular adhesion molecules (molecules that allow white blood cells to adhere to the endothelium) (i.e., VCAM-1, ICAM-1, and ELAM-1) on the endothelial cell surface (Grilli et al. 1993). In turn, these cellular adhesion molecules help recruit a special type of white blood cell (i.e., mononuclear leukocytes, or monocytes) to the vascular endothelium. Recruited monocytes adhere to the endothelium, then move into the subendothelial space and differentiate into macrophages that engulf (i.e., phagocytose) oxidized LDL, forming “foam cells.” The foam cells, as well as other cells, subsequently produce intercellular mediators (i.e., cytokines) and growth factors that induce cell proliferation. Blood platelets are activated, and a blood clot (i.e., thrombus) forms at the inflammatory site.

Several theories have been suggested to explain how alcohol possibly thwarts this inflammatory process and provides protection against atherogenesis. For example, alcohol may disrupt the NF-κB function, thereby reducing the expression of NF-κB–regulated adhesion molecules and ultimately decreasing the inflammatory process of atherosclerotic lesions.

From another angle, the hypothesis that LDL oxidation leads to the formation of fatty streaks prompted the speculation that antioxidants in wine may contribute protective effects. Red wine and, to a lesser extent, other alcoholic beverages contain several flavonoids and phenolic compounds with significant antioxidant properties. However, de Rijke and colleagues (1996) concluded that the cardioprotective effects associated with red wine are unlikely to result solely from the antioxidant properties of these compounds, because the quantity of antioxidants consumed in wine may not reach sufficiently high plasma levels to prevent the oxidation of LDL. On the other hand, Abu-Amsha and colleagues (1996) found that the phenolic compounds present in both alcoholic and nonalcoholic beverages protected against LDL oxidation. Furthermore, Pellegrini and colleagues (1996) observed that drinking dealcoholized red wine for 4 weeks changed the composition of blood platelets and decreased the likelihood of blood clot formation.

**Figure:**
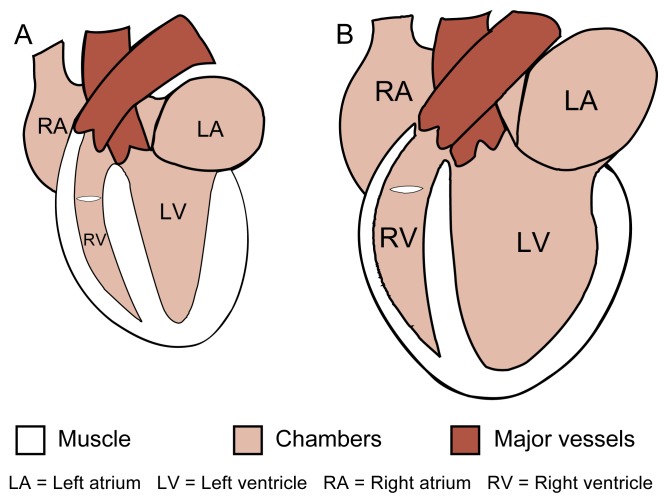
Schematic illustration of a normal heart (A) and a heart in dilated cardiomyopathy (B). Both hearts are shown in their state at the end of contraction (i.e., at endsystole).

HDL also inhibits LDL oxidation. This effect is attributed to two enzymes associated with HDL: platelet-activating factor acetylhydrolase and paraoxonase. Although alcohol has been shown to increase HDL levels, as previously discussed, no data are available to show how alcohol might influence these two enzymes in particular.

Another possible mechanism for CAD risk reduction relates to the inhibition of cell proliferation that results from cellular signaling. Blocking the action of the enzyme known as hydroxymethylglutaryl coenzyme A (HMG-CoA) reductase has been shown to suppress cell proliferation, among other effects (e.g., cholesterol reduction), and thus may provide another way to reduce plaque formation and slow atherosclerosis development. Certain cholesterol-lowering medications (e.g., lovastatin) block HMG-CoA reductase, but further research is needed to determine whether alcohol has a similar effect.

### Alcohol’s Effects on Platelet Function in Blood Clot Formation

As plaque builds up within the wall of an artery, the deposit begins to bulge into the vessel’s interior, obstructing blood flow, and eventually may rupture into the vessel. When a rupture occurs, platelets coming in contact with collagen and other exposed subendothelial compounds become activated and operate in conjunction with other clotting (i.e., coagulation) factors to form a blood clot and seal off damage.

Platelet function is a key factor in the initiation and progression of blood clot formation (i.e., thrombosis). This complex process begins in the roughened endothelial surface of a damaged blood vessel. Platelets adhere to the roughened surface, and a cascade of events culminates in the formation of the enzyme thrombin. Thrombin interacts with platelet membrane receptors, resulting in stimulation of the enzyme phospholipase C. This enzyme mediates platelet aggregation through the formation of two compounds, inositol triphosphate and diacylglycerol. The former compound mobilizes ionized calcium from intracellular stores, and the latter activates another enzyme known as protein kinase C. Both calcium and protein kinase C induce two critical steps in the clotting process—platelet aggregation and release of the platelets’ granular contents—that in turn activate additional platelets. In addition, calcium and protein kinase C stimulate platelets to form a compound known as thromboxane A_2_, which also acts as a powerful stimulator of platelet aggregation and activation.

Once a blood clot forms, it may eventually clog the vessel or break off (becoming an “embolus”) and lodge elsewhere in the circulatory system. In either case, the results are potentially serious. For example, a thrombus or embolus interrupting the blood supply to the heart could produce a heart attack (i.e., myocardial infarction), whereas a blood clot impairing the blood supply to the brain could cause a stroke. Thus, any factor that reduces platelet aggregation, inhibits blood clot formation, or promotes blood clot dissolution (discussed in the next section) could attenuate the thrombotic complications of atherosclerosis. Moderate alcohol consumption may ameliorate all of these processes, which would help explain the antithrombotic effects of alcohol reported by several researchers.

At moderate consumption levels, McKenzie and Eisenberg (1996) found that alcohol did not impair the normal synthesis of coagulation factors. Instead, alcohol’s antithrombotic effects appear to be related to platelet granule secretion and inhibition of thromboxane A_2_ production. Alcohol also may affect the structural integrity of activated platelets by interfering with granule fusion, which results in changes in platelet shape (Stubbs and Rubin 1992). When alcohol consumption is chronic, platelet function is significantly reduced, and clotting time increases. Even after alcohol intake ceases, these effects persist for several weeks.

### Alcohol’s Effects on Blood Clot Dissolution

Normally, a balance exists between the compounds involved in blood clot formation and dissolution (i.e., fibrinolysis). Plasmin, the enzyme responsible for degrading the clot component fibrin and its precursor (i.e., fibrinogen), circulates in the form of its inactive precursor (i.e., plasminogen) until produced by plasminogen activators (i.e., tissue-type plasminogen activator [t-PA] and urokinase-type plasminogen activator [u-PA]). The principal physiologic inhibitor, plasminogen activator inhibitor type-1 (PAI-1), terminates the fibrinolytic process.

Epidemiological studies have reported a positive association between alcohol consumption and fibrinolytic activity in men and women. Iso and colleagues (1993) reported significant increases in plasma t-PA levels in heavy drinkers, and a recent study by Hendriks and colleagues (1994) also showed a sustained increase in t-PA following moderate consumption of alcohol with dinner. An increase in the plasma level of t-PA presumably would stimulate the conversion of plasminogen to its active form, plasmin; in turn, raising the level of plasmin would increase blood clot dissolution.

In addition to epidemiological studies, in vitro studies have investigated the effects of alcohol on fibrinolysis. Laug (1983) reported alcohol-induced increases in t-PA secretion in cultured endothelial cells, and Kjaeldgaard and colleagues (1988) observed similar effects with a human melanoma cell line. Reeder and colleagues (1996) suggested that the interaction between alcohol consumption and fibrinolysis may involve the influence of rhythmic daily fluctuations in the levels of fibrinolytic proteins, but the exact mechanisms remain to be elucidated.

A study by Ridker and colleagues (1994) provided further evidence that alcohol induces the secretion of t-PA. Ironically, however, although secretion of this protein may contribute to the protective effect of alcohol against adverse coronary events, it also may increase alcohol-induced risk of hemorrhage, because heightened fibrinolytic activity will hinder the ability of blood clots to stop internal bleeding if a blood vessel ruptures.

An additional factor in alcohol’s perturbing effect on fibrinolytic proteins may involve its effects on modifiers that influence fibrinolytic activity, such as the serum level of triglycerides. An increase in triglyceride level is positively correlated with PAI-1 plasma levels, indicating a predisposition to thrombosis and atherogenesis (Reeder et al. 1996). Moderate alcohol consumption decreases fasting plasma concentrations of triglycerides, however, and a concomitant reduction in the level of PAI-1 could allow fibrinolytic activity to increase. In contrast, heavy alcohol consumption may have the opposite effect. Elevated triglyceride levels resulting from heavy alcohol consumption may further stimulate PAI-1 gene expression—especially in people with a genetic makeup particularly sensitive to PAI-1—resulting in the inhibition of fibrinolysis and thus increasing the risk for acute cardiac events.

## Cardiovascular Consequences of Heavy Alcohol Consumption

Alcohol-induced damage to the cardiovascular system may result from either excessive prenatal alcohol exposure or from excessive alcohol use later in life. Fetal exposure produces a variety of congenital cardiovascular malformations. This article, however, focuses on four specific cardiovascular consequences (i.e., cardiomyopathy, cardiac arrhythmia, hypertension, and stroke) that result from heavy drinking later in life.

### Alcohol and Cardiomyopathy

A condition known as dilated cardiomyopathy constitutes a major subset of the disorders grouped under the umbrella term “cardiomyopathy,” which encompasses any chronic disorder affecting the heart muscle. Dilated cardiomyopathy is characterized by low cardiac output and enlargement of the heart (i.e., hypertrophy) and its chambers (i.e., dilatation) (see figure) and eventually leads to congestive heart failure (CHF). A variety of factors can cause dilated cardiomyopathy, including prolonged heavy drinking (i.e., alcoholic cardiomyopathy) as well as the late effects of viral infections and toxic substances. Although alcoholic cardiomyopathy may be reversible after abstention, severe cases still may progress into CHF despite a cessation of alcohol use.

The association between excessive alcohol consumption and enlargement of the heart and the occurrence of CHF in chronic alcoholics was first reported more than 100 years ago. More recent research has further established the association between cardiomyopathy and heavy alcohol consumption (Moushmoush and Abi-Mansour 1991; Rubin and Thomas 1992). Alcoholic cardiomyopathy accounts for 20 to 50 percent of all cases of cardiomyopathy in Western countries. In addition, many alcoholics exhibit some degree of subclinical depression of cardiac function. Although cardiomyopathy is more common in men than women, a recent study showed that women are actually more susceptible to alcohol-induced cardiomyopathy than men (Urbano-Marquez et al. 1995). In that study, the prevalence of cardiomyopathy in men and women was comparable, even though the total lifetime alcohol consumption by women was only 60 percent that of men.

Animal models do not replicate human cardiomyopathy exactly, but they can provide insight into the mechanisms of alcohol-induced damage. The hearts of animals fed alcohol for several months exhibited depressed contractile function. For example, dogs fed alcohol for 1 year and rats fed alcohol for 8 months showed significant decreases in left ventricular function (Capasso et al. 1991).

Several mechanisms have been identified to explain alcohol’s negative effects on cardiac muscles. For example, when an electrical current spreads to the interior of cardiac muscle fibers, it causes the release of large quantities of calcium ions from a network of tubules (i.e., the sarcoplasmic reticulum), which in turn trigger the chemical events that produce muscular contractions. Alcohol alters the permeability of the sarcoplasmic reticulum to calcium ions, however, and thus reduces the efficiency by which calcium activates muscle contraction (Thomas et al. 1996). In addition, alcohol has a negative effect on the integrity and function of the contractile proteins known as actin and myosin (Preedy et al. 1996). Alcohol reduces the synthesis of cardiac proteins in both the contractile apparatus (i.e., the actinmyosin complex) and in the cell’s “powerhouses” (i.e., mitochondria), especially in alcoholics with high blood pressure (Preedy et al. 1996). Similarly, acetaldehyde (a metabolite of alcohol) and free radicals may contribute to decreased protein synthesis as well. Another way that alcohol can induce cardiac muscle damage is by increasing the expression of a certain gene (i.e., *c-myc*), which can promote programmed cell death, resulting in muscle cell loss (Paice et al. 1996).

The biochemical basis of alcohol-induced cardiomyopathy also involves disturbances in cardiac energy metabolism. For example, high blood concentrations of alcohol reduce the oxygen supply to the cardiac muscle and interfere with oxygen-requiring (i.e., aerobic) metabolism in the heart. This effect decreases the level of the high-energy molecules that power the contraction process (i.e., adenosine triphosphate [ATP]) as well as the level of another energy source, phosphocreatine.

### Alcohol and Cardiac Arrhythmias

Electrophysiological changes in cardiac rhythm have been described after episodes of substantial acute alcohol intake as well as chronic alcohol consumption. For example, acute disturbances in cardiac rhythm following heavy alcohol consumption over a long weekend—generally referred to as “holiday heart syndrome”—are characterized by specific electrocardiographic changes that are hallmarks of cardiac conduction abnormalities.

Disturbed ventricular cardiac rhythms (i.e., ventricular arrhythmias) associated with sudden cardiac death also have been attributed to alcohol abuse. In a Finnish study, 5.2 percent of deaths from ventricular arrhythmias among 15- to 49-year-olds were attributed to alcoholism. Furthermore, a case-controlled study of sudden death in middle-aged women attributed one-half of these deaths to alcoholism.

Alcohol abuse also can cause rapid and chaotic heartbeats to occur in the upper chambers of the heart (i.e., atrial fibrillation), although numerous other risk factors (e.g., age, hypertension, CAD, and diseases of the heart valves) can precipitate this condition as well. Most cases of atrial fibrillation are caused by factors other than alcohol, but one study reported that the majority of emergency room patients with atrial fibrillation had a history of alcohol abuse (Rich et al. 1985). In research comparing people who consumed less than one alcoholic beverage per day with drinkers who consumed six or more drinks daily, the latter group experienced a twofold increase in risk for atrial fibrillation, atrial flutter (a less severe arrhythmia than atrial fibrillation), premature beats in the upper chambers of the heart (i.e., the right and left atria), and supraventricular tachycardia (i.e., an increased rate of contraction in the atria).

Several mechanisms for alcohol-induced cardiac arrhythmias have been proposed. For instance, increased thickening and scarring of connective tissue (the tissue between cardiac cells) in heart muscle, which has been observed in alcoholic cardiomyopathy, could provide the anatomical source of the disturbance in ventricular rhythm by impeding electrical conduction. Alcohol-induced arrhythmias also may be caused by a reduction in the threshold for ventricular fibrillation. Other mechanisms include electrolyte disturbances, a lack of oxygen to the heart muscle, and an increase in basal plasma levels of the substances involved in transmitting impulses from nerves to muscles (i.e., catecholamines).

### Alcohol and Hypertension

Numerous epidemiological studies have established an association between chronic alcohol consumption and hypertension independent of other risk factors such as obesity and smoking, and their results have been summarized previously (Beilin and Puddey 1992; Klatsky 1995; Camargo and Rimm 1996). This association has been observed with alcohol consumption in excess of two drinks per day and described in white, black, and Asian men and women who reported daily intake of three or more drinks (see, for example, Klatsky 1995). Women may be less susceptible than men to alcohol-induced hypertension, however.

Chronic alcohol consumption has been verified as the cause of hypertension in two controlled trials. In the first study, the blood pressure of 16 hypertensive men, who drank 4 pints of beer on average, dropped significantly when alcohol was withdrawn for 4 days (Potter and Beevers 1984). In the second study, 20 hypertensive subjects (10 who reported consuming less than 2 drinks per day and 10 who reported consuming 2 to 6 drinks per day) showed significant blood pressure reductions after abstinence (Malhotra et al. 1985). Intervention studies also showed that consumption of three to eight alcoholic beverages per day by subjects whose blood pressure was within or above the normal range (i.e., normotensive and hypertensive subjects, respectively) increased blood pressure and that either total abstinence from alcohol or a reduction to less than one drink per day resulted in a short-term drop in blood pressure.

An important aspect of the alcohol-hypertension association—and a fertile area for future studies—concerns alcohol’s interactions with antihypertensive medications such as propranolol and clonidine. Alcohol enhances the elimination of propranolol and opposes the effect of clonidine, resulting in a decrease in the blood pressure-lowering properties of these medications. Also, because chronic alcohol consumption decreases the concentration of magnesium ions in the blood, the use of medications that increase kidney excretion of electrolytes and water (i.e., diuretics) to control blood pressure may be contraindicated, because their use can exacerbate magnesium loss.

Several mechanisms have been advocated to explain how alcohol induces hypertension, including the following:

- Increased activity of the sympathetic nervous system (Russ et al. 1991). The sympathetic nervous system plays a major role in cardiovascular regulation by constricting blood vessels and increasing the contractile force of the heart, thus raising blood pressure.
- Increased plasma levels of compounds involved in transmitting impulses from nerves to muscles (i.e., catecholamines). Catecholamines (e.g., adrenaline and noradrenaline) help maintain blood pressure and will cause hypertension if present in excess.
- Decreased sensitivity of the baroreceptors located in artery walls (El-Mas and Abdel-Rahman 1993). Baroreceptors normally respond to the stretching that results from a rise in blood pressure by transmitting signals to the central nervous system. In response, the central nervous system returns signals to the circulatory system to reduce blood pressure toward normal.
- Decreased levels of electrically charged (i.e., ionized) magnesium in plasma (Altura and Altura 1994). A delicate balance between magnesium and calcium ions maintains vascular tone. Magnesium ions cause blood vessels to relax, whereas calcium ions have the opposite effect and cause them to constrict. If the level of magnesium ions is reduced (i.e., the ratio of ionized magnesium to ionized calcium is reduced), then ionized calcium will predominate, causing an increase in blood pressure as the vessels constrict.

### Alcohol and Stroke

Stroke is an acute cerebrovascular disorder that encompasses two major types: ischemic stroke, in which plaque buildup or a blood clot impairs blood flow to the brain, and hemorrhagic stroke, in which a ruptured artery interrupts blood from reaching the brain. Ischemic stroke comprises 80 percent of stroke cases, whereas hemorrhagic stroke accounts for the remaining 20 percent.

Ischemic stroke occurs most commonly in elderly people, and its major risk factors are hypertension and smoking. Alcohol’s relationship to ischemic strokes involves interactions with several cardiovascular problems (e.g., CAD, hypertension, rhythm disturbances, and cardiomyopathy). Nonetheless, light-to-moderate alcohol consumption seems to reduce the risk of ischemic strokes caused by plaque buildup (i.e., atherosclerotic ischemic strokes) (Palomäki et al. 1993), and factors that could explain this reduction include the following:

- Inhibition of LDL oxidation, thus reducing the development of atherosclerosis
- Inhibition of thromboxane A_2_ release, which would have an antithrombotic effect
- Increased formation of a compound known as prostacyclin, which is the most potent inhibitor of platelet aggregation known
- Decreased platelet activity (Renaud et al. 1992)
- Increased plasma levels of t-PA, which promotes dissolution of the blood clots that may precipitate a stroke (Hendriks et al. 1994).

Heavy alcohol consumption, on the other hand, has precipitated ischemic strokes caused by blood clots (i.e., non-atherosclerotic, or emoblic, ischemic strokes) (Hillbom 1995). The increase in embolic stroke in heavy drinkers has been attributed to atrial fibrillation and cardiomyopathy, because both of these conditions can predispose a person to either the formation of blood clots or the propagation of existing clots that could ultimately dislodge and block blood flow to the brain (Qureshi et al. 1995).

Regarding the less common hemorrhagic type of stroke, alcohol also has been associated with an increased risk of bleeding within the cerebrum (i.e., intracerebral hemorrhage) and, less frequently, within the space surrounding the entire brain and spinal cord (i.e., the subarachnoid space). In 1995 Iso and colleagues reported that chronic heavy drinkers have at least twice the risk of intracerebral hemorrhage of nondrinkers. In another study, intracerebral hemorrhage was associated with alcohol abuse in 28 percent of the cases (Qureshi et al. 1995). Alcohol-induced intracerebral hemorrhage was more pronounced in hypertensive than normotensive subjects (Kiyohara et al. 1995), indicating that alcohol-induced hypertension may predispose a drinker to this type of stroke. Hypertension also could play a role in alcohol-induced subarachnoid hemorrhage.

Both chronic heavy drinkers and binge drinkers are at an increased risk for subarachnoid hemorrhage. One study attributed 12 percent of subarachnoidal hemorrhage cases to recent heavy drinking (Juvela et al. 1993). Other research suggested that such cases could be precipitated by a transient increase in blood pressure. Smoking also is an important risk factor for subarachnoid hemorrhage (Juvela et al. 1993), and the combined effects of heavy drinking and smoking may be devastating.

Ironically, the antithrombotic effects of alcohol, although beneficial in reducing the risk of CAD, could play an important role in increasing the risk of hemorrhagic strokes, because inhibited blood clot formation would result in more severe bleeding if an artery ruptures. Women appear to be especially sensitive to an increased risk of hemorrhagic stroke, even at the relatively low levels of alcohol consumption associated with protection against ischemic stroke (Fuchs et al. 1995).

## Conclusion

The ancient Roman god Janus is depicted with two faces looking in opposite directions. Like the faces of Janus, alcohol’s effects on the cardiovascular system can take opposite forms, depending on how much is consumed, by whom, when, and how. Numerous epidemiological studies have concluded that light-to-moderate drinking is associated with improved cardiovascular health. Certain people who are at higher risk for CAD (e.g., men over age 45, postmenopausal women, and smokers) can benefit from the cardioprotective effects of alcoholic beverages to a greater extent than people without such risk factors. The grim face of Janus, however, represents the toxic effects that chronic heavy alcohol consumption has on the cardiovascular system. Many studies on humans and animals have documented negative effects, including cardiomyopathy, cardiac arrhythmias, hypertension, and stroke.

Significant progress has been made in the last decade in understanding both the beneficial and harmful effects of alcohol on the cardiovascular system. Nevertheless, alcohol has numerous secrets that remain to be uncovered by ongoing research. Further investigations will clarify some of the effects of alcohol discussed in this article (e.g., its effects on fibrinolysis), including its mechanisms (e.g., alcohol’s possible role in the inhibition of the enzyme HMG-CoA reductase). In addition, other atherogenesis-related factors (e.g., the influence of hormones) that are not addressed in this article may be influenced by the use of alcohol, and research is needed to investigate these factors as well.

## Footnotes

1 The term “heavy drinking” is not used consistently in the alcohol literature; therefore, this article generally refers to “heavy drinking” and “heavy drinkers” based on the terms used in the reference cited.

2 The terms “alcoholism” and “alcoholic” as used in this article are summary terms for the diagnoses of alcohol abuse and alcohol dependence.

3 In this article, the term “moderate drinking” generally refers to the consumption of one or two drinks per day. Moderate drinking cannot be achieved by simply averaging the number of drinks consumed, however. For example, consuming seven drinks on a Saturday night will not have the same effects as consuming one drink each day of the week.

4 For example, Psalm 104:14–15 mentions “wine that gladdens the heart of man” as one of the fruits brought forth from the earth as a result of God’s handiwork, and in I Timothy 5:23, Paul advises Timothy: “Stop drinking only water, and use a little wine because of your stomach and your frequent illnesses.”

5 For a definition of this and other technical terms, see the glossary, pp. 93–96

6 Alcohol also may affect LDL and triglyceride levels, but its influences on these substances are beyond the scope of this article (for more information, see the exhaustive review by Dreon and Krauss 1996).
